# How brain asymmetry relates to performance – a large-scale dichotic listening study

**DOI:** 10.3389/fpsyg.2013.00997

**Published:** 2014-01-02

**Authors:** Marco Hirnstein, Kenneth Hugdahl, Markus Hausmann

**Affiliations:** ^1^Department of Biological and Medical Psychology, University of BergenBergen, Norway; ^2^Division of Psychiatry, Haukeland University HospitalBergen, Norway; ^3^Department of Radiology, Haukeland University HospitalOslo, Norway; ^4^Department of Psychology, Durham UniversityDurham, UK

**Keywords:** hemispheric asymmetry, lateralization, dichotic listening, task-performance, sex, age, handedness, verbal abilities

## Abstract

All major mental functions including language, spatial and emotional processing are lateralized but how strongly and to which hemisphere is subject to inter- and intraindividual variation. Relatively little, however, is known about how the degree and direction of lateralization affect how well the functions are carried out, i.e., how lateralization and task performance are related. The present study therefore examined the relationship between lateralization and performance in a dichotic listening task for which we had data available from 1839 participants. In this task, consonant-vowel syllables are presented simultaneously to the left and right ear, such that each ear receives a different syllable. When asked which of the two they heard best, participants typically report more syllables from the right ear, which is a marker of left-hemispheric speech dominance. We calculated the degree of lateralization (based on the difference between correct left and right ear reports) and correlated it with overall response accuracy (left plus right ear reports). In addition, we used reference models to control for statistical interdependency between left and right ear reports. The results revealed a u-shaped relationship between degree of lateralization and overall accuracy: the stronger the left or right ear advantage, the better the overall accuracy. This u-shaped asymmetry-performance relationship consistently emerged in males, females, right-/non-right-handers, and different age groups. Taken together, the present study demonstrates that performance on lateralized language functions depends on how strongly these functions are lateralized. The present study further stresses the importance of controlling for statistical interdependency when examining asymmetry-performance relationships in general.

## INTRODUCTION

Beginning with the discovery of the left-hemispheric dominance of language (Broca, 1861; Dax, 1865) it has now been shown that practically all higher functions including memory, learning, perception, spatial cognition, attention, complex motor skills, and emotion processing show some degree of hemispheric specialization (Hellige, 1993; Davidson and Hugdahl, 1995). At first, lateralization was believed to be a unique human feature (Crow, 2002) but in the meantime it has been documented in a wide range of species (Vallortigara and Rogers, 2005). Brain asymmetries in humans, however, are typically more pronounced than in animals and it has been argued that they gave rise to our superior verbal and intellectual skills (Corballis, 1991, 2009). Previous research has shown that the degree of lateralization in humans is subject to inter- and intraindividual differences. For example, some individuals show strong left-hemispheric language lateralization, others strong-right-hemispheric language lateralization, and still others possess a more bilateral language representation (Knecht et al., 2000). Even within individuals lateralization changes as a function of, for example, sex hormones (Hausmann and Güntürkün, 2000; Bayer and Hausmann, 2009; Hjelmervik et al., 2012) or emotional states (Papousek et al., 2011, 2012). However, not much is known about how degree of lateralization and performance in selected functions are related, which we refer to as the “asymmetry-performance relationship”, and the few studies available provide incoherent results. For example, Everts et al. (2009) found that a stronger language lateralization, determined with functional magnetic resonance imaging (fMRI), was correlated with a higher verbal IQ. Chiarello et al. (2009) used visual half-field paradigms to assess language lateralization and also found a positive correlation between the degree of lateralization in these tasks and reading skills. On the other hand, there are also studies showing that performance deteriorates with increasing asymmetry. For example, less lateralized participants outperform more lateralized individuals in a face discrimination task (Ladavas and Umilta, 1983) and when two cognitive tasks (i.e., face discrimination and lexical decision) are performed in parallel (Hirnstein et al., 2008). Moreover, individuals with higher degrees of language lateralization as determined with fMRI (van Ettinger-Veenstra et al., 2010) or magnetic resonance diffusion tensor imaging (Catani et al., 2007) performed better on tests assessing verbal abilities (van Ettinger-Veenstra et al., 2010) or verbal memory (Catani et al., 2007) than individuals with lower degrees of lateralization. The inconsistent findings are neatly illustrated by Razafimandimby et al. (2011) who found that verb generation correlated both positively with precuneus asymmetry and negatively with cerebellum asymmetry (as determined with fMRI).

Boles et al. (2008) carried out the most extensive investigations regarding the asymmetry-performance relationship. They had data from several visual half-field and dichotic listening (DL) tasks that assessed various verbal and non-verbal cognitive functions. To obtain the asymmetry-performance relationship, they correlated the degree of lateralization derived from these tasks with the overall accuracy (or reaction times) – also derived from these tasks. The results are in line with the inconsistent findings described above. Boles et al. (2008) found positive asymmetry-performance relationships in four tasks assessing auditory linguistic and spatial positional functions. Negative relationships emerged in seven tasks assessing planar categorical, spatial emergent, spatial quantitative, and visual lexical functions. The authors concluded that the asymmetry-performance relationship is function-dependent and suggested a neurodevelopmental model according to which functions that lateralize very early (until 5 years of age) and very late in the ontogenetic development (after 11 years of age) yield positive asymmetry-performance correlations. Functions that lateralize at intermediate stages on the other hand show negative correlations.

The neurodevelopmental theory of Boles et al. (2008) may account for some of the strikingly inconsistent results. However, there are a number of methodological pitfalls which might contribute to the inconsistencies above. One of these issues is the “task purity problem” (Boles and Barth, 2011). If lateralization is assessed with one task and then correlated with performance in another task, correlations between lateralization and performance might be confounded by a third variable and do not reveal the pure asymmetry-performance relationship (Boles and Barth, 2011; but see also the reply of Chiarello et al., 2011). If one derives the performance and lateralization from the same task, however, one is faced with the problem of interdependency between left (L) and right (R) scores. Both the overall accuracy (i.e., sum or mean of L and R) *and* the degree of lateralization [i.e., (R - L)/(R + L) or (R - L)/(200 - R - L)] are derived from the same L and R scores. Given that L and R scores are typically correlated with each other, there is a risk that the asymmetry-performance relationship is simply the result of, or at least confounded with, this correlation between L and R scores.

The vast majority of studies that investigated the asymmetry-performance relationship in one task do not address the interdependency issue. To solve this problem, Leask and Crow (1997, 2006) developed a method that compares the asymmetry-performance relationship based on R and L scores with reference models in which R and L scores have been modeled such that they do not correlate. Another advantage of this procedure is that it is data-driven and can detect any form of asymmetry-performance relationships. Most studies simply assume linear asymmetry-performance relationships. By applying the procedure suggested by Leask and Crow (1997, 2006) to data from two visual half-field paradigms (i.e., word recognition, face discrimination), Hirnstein et al. (2010) found an inverted u-shaped association between asymmetry and performance. That is, individuals with a symmetric brain organization performed best and performance deteriorated with increasing left or right lateralization. However, the calculation of the degree of asymmetry [(R - L)/(R + L)] in this study has been criticized by Boles and Barth (2011).

It should be noted that almost all of the aforementioned studies that investigated the asymmetry-performance relationship tested right-handed adults (Catani et al., 2007; Boles et al., 2008; Hirnstein et al., 2010; van Ettinger-Veenstra et al., 2010) leaving it unclear whether the findings also apply to other populations such as left-handers, children and adolescents, which are assumed to be less lateralized in verbal and non-verbal functions (e.g., Rasmussen and Milner, 1977; Everts et al., 2009). In general, interindividual differences in the asymmetry-performance relationship are hardly investigated even though there are hints that they exist. Chiarello et al. (2009) reported that the positive correlation between language lateralization and reading skills was stronger in individuals with a consistent hand preference as compared to participants with an inconsistent hand preference. Hirnstein et al. (2010) found that males with a strong left-hemispheric lateralization in a face discrimination task performed rather poorly, while females with a strong left-hemispheric lateralization performed rather well. Thus the asymmetry-performance relationship might also be sex-specific. Finally, little is known about age effects. Only Barth et al. (2012) studied whether the positive asymmetry-performance relationship that they found in a verbal DL task in adults (Boles et al., 2008) also emerged in children. Moreover, they examined whether, in accordance with their neurodevelopmental model, adults but not children showed a negative relationship in emotional face discrimination. While the results mostly confirmed their hypotheses, some of the correlations did not reach statistical significance. According to the authors this was due to the relatively small sample size (25 children, 32 adults) emphasizing that sufficient statistical power is needed to reveal the asymmetry-performance relationship.

With some exceptions (Boles et al., 2008; van Ettinger-Veenstra et al., 2010; Barth et al., 2012) most of the studies on the asymmetry-performance relationship used visual tasks and visual asymmetry (Ladavas and Umilta, 1983; Boles et al., 2008; Hirnstein et al., 2008, 2010; Chiarello et al., 2009). Since the relationship between brain asymmetry and task performance should be generic and not dependent on sensory modality, similar relationships should be possible to obtain in the auditory modality, using, e.g., a DL task, which is perhaps the most frequently used task for assessing hemispheric asymmetry (see Hugdahl, 2011; Kimura, 2011 for recent overviews of the use of DL in asymmetry research). Over the years, Kenneth Hugdahl and our research group at the University of Bergen have built up a database with DL data, which now comprises 1839 individuals (see Hugdahl, 2003 for a description of the database). The sample covers a wide age range (5–89 years), has a balanced sex ratio (927 females, 912 males) and a proportion of non-right-handers of 8.9% which is close to the 10% typically observed in the general population (McManus, 2002). The large number of participants allows a comprehensive examination of the asymmetry-performance relationship and further provides an ideal opportunity to also take into account sex, handedness, and age effects. Two previous studies found that overall accuracy in verbal DL increased as asymmetries became stronger (Boles et al., 2008; Barth et al., 2012), however, leaving the interdependency issue of L and R scores unsolved. Using the approach by Boles et al. (2008), the present study examined first whether we could replicate the positive asymmetry-performance relationship found by this group. In a second step, we applied the approach by Leask and Crow (1997, 2006) which controls for the interdependency issues. By applying this approach, we also took sex, handedness, and age into account. In line with Boles et al. (2008), we hypothesized that individuals with stronger ear advantages (corresponding to a stronger degree of language lateralization) would generally report more stimuli correctly. Consequently, non-right-handers, women, and children, who are assumed to be less lateralized for language, should generally report less syllables correctly. However, this requires asymmetry-performance relationships to be consistent across all subsamples.

## MATERIALS AND METHODS

### PARTICIPANTS

All 1839 participants in the database completed the DL task described below. The database includes data that have been collected by collaborators in many countries, laboratories, and clinics. They all used the same stimulus materials (but in their native language) and procedure for administering the task, specified in a manual prepared by the Bergen group and distributed to collaborators. The database comprises native Norwegian, Swedish, Finnish, English, German, Slovak-, and Spanish speaking individuals. Handedness was assessed with either the Edinburgh Handedness Inventory (Oldfield, 1971) or the Raczkowski questionnaire (Raczkowski et al., 1974). Participants were classified as right- or left-handed, if they preferentially carried out the majority of actions in these questionnaires with the right or left hand, respectively. Seven participants in the database had been coded as ambidexters (0.4%). Since this group was too small for any meaningful statistics, these participants were collapsed with the left-handers into a “non-right-handers” group. When the database was set up many years ago, age was not considered a major variable and participants were only allocated to age groups. Later, the exact age was included additionally. As a result, the exact age is known for 993 participants (54%), but *all* participants had been allocated to one of these groups: children (5–9 years), early adolescents (10–15 years), younger adults (16–49 years), and older adults (≥50 years). The boundary of 16 was chosen as it was, and still is, the lower limit of the Wechsler Adult Intelligence Scale (Wechsler, 2008). The other boundaries were chosen such that the number of participants was fairly balanced in each category by the time the database was set up. In the interest of statistical power we thus used the existing four group system. An overview of the sample with exact numbers of participants across the factors sex, handedness, and age is provided in **Table 1**.

**Table 1:** Number of participants in the Bergen DL database across age, sex, and handedness.

		Children (5–9 years)	Early adolescents (10–15 years)	Younger adults (16–49 years)	Older adults (≥50 years)	Σ
Females	Right-handed	99	208	434	101	842
	Non-right-handed	3	9	68	5	85
Males	Right-handed	100	293	353	88	834
	Non-right-handed	9	24	41	4	78
Σ		211	534	896	198	1839

The database comprises participants without known hearing deficits, psychiatric and neurological disorders. The majority of participants had been assessed with a hearing threshold test. All of them were able to detect frequencies of up to 3000 Hz at an intensity of 20 dB and the interaural acuity difference was ≤10 dB.

### STIMULUS MATERIAL AND PROCEDURE

The Bergen DL task has been validated as a measure of language lateralization with ^15^O positron emission tomography (Hugdahl et al., 1999) and the sodium-amytal test (Hugdahl et al., 1997). The task consists of six consonant-vowel syllables (/ba/, /da/, /ga/, /pa/, /ta/, /ka/). For each trial, two syllables are presented at the same time via headphones – one syllable to the left and the other to the right ear. All possible 36 combinations of the six syllables are presented once in a pseudo randomized order, including the six homonyms (e.g., /ba/ /ba/) which were not used in the statistical analysis. The intertrial interval was about 4 s. The syllables are temporally aligned to ensure simultaneous onset of the consonant segment and the mean stimulus duration is around 350–450 ms depending on voice onset time differences between unvoiced and voiced consonants and on the language. The stimuli were presented at a sound intensity of about 70 dB (with slight variations between laboratories and clinics). Again, depending on the laboratory and clinic, stimuli were presented PC-based or via analog or digital tape/CD players. The participants were not informed that there were two different syllables at each trial and their instruction was to report one syllable – the one they heard best and most clearly. Participants were tested with syllables in their respective mother tongue. For instance, native Norwegian speakers completed the task with syllables spoken by a native Norwegian speaker, German participants with syllables spoken by a native German speaker, etc. The syllables were spoken by a male voice with constant intensity and intonation for all languages. The dependent variable was the number of correctly reported syllables for each ear (maximum correct reports = 30 in total).

## DATA ANALYSIS

### TRADITIONAL APPROACH Boles et al. (2008)

To compare our data with previous DL findings (Boles et al., 2008), we first used the traditional approach of simply correlating overall accuracy and degree of lateralization. The overall accuracy was determined as the sum of R and L scores, with R and L corresponding to the percentage of correctly reported syllables from the right and left ear, respectively. To determine the degree of lateralization we calculated a laterality coefficient (LC) using the formula [(R - L)/(R + L)] × 100. Positive values thus reflect a right ear/left-hemispheric advantage while negative values correspond to a left ear/right-hemispheric advantage for language perception. This formula was chosen because the Bergen DL Task is a one-response paradigm. That is, in each trial participants report either the left *or* the right ear stimulus depending on which one they perceive best. This is different to two-response paradigms, in which participants are instructed to report all stimuli (i.e., from the left *and* the right ear). In two-response paradigms, accuracy rates for both ears can add to 100% and the mean accuracy across both ears can thus also be 100%. In one-response paradigms, however, only one ear can obtain an accuracy rate of 100% and the mean accuracy can never exceed 50%. Therefore the practice of using two formulas in two-response paradigms (one for mean accuracies above 50% and another for mean accuracies below 50%) does not apply to our paradigm (cf. Repp, 1977).

The overall accuracy and the LC were entered as dependent and independent variables, respectively, in linear and quadratic regressions. Quadratic regressions were computed to test potential u-shaped asymmetry-performance relationships (Leask and Crow, 2006; Hirnstein et al., 2010). Moreover, linear and quadratic regressions were carried out for absolute LC values in order to investigate the relationship between performance and the *strength* of lateralization regardless of direction.

### LOESS APPROACH (Leask and Crow, 2006)

**FIGURE 1:**
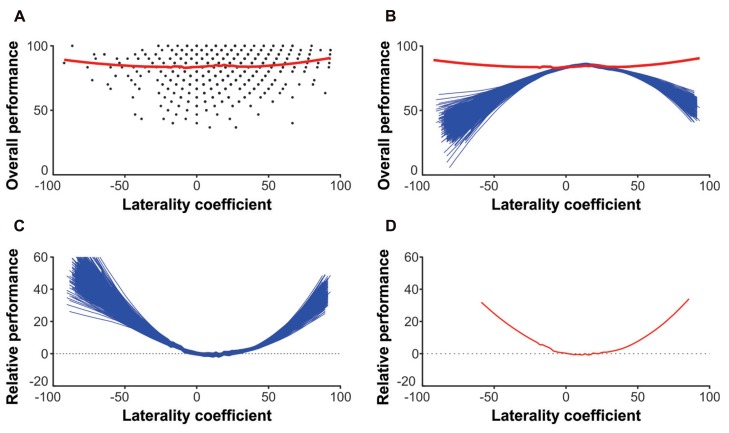
**Results and principle of the LOESS method. (A)** The raw left and right ear reports are used to compute the laterality coefficient and the overall accuracy. Laterality coefficient and overall accuracy are fitted with LOESS (red line). **(B)** Reference models (blue lines) are computed which are based on the raw left and right ear reports but not correlated (*r* < 0.01). The reference models are also fitted with LOESS. **(C)** The reference models are subtracted **(C)** from the raw data and then averaged **(D)** to reveal the asymmetry-performance relationship controlled for interdependency.

The general principle of the alternative approach is to compare the original data with a reference model in which the interdependency has been removed. The procedure is illustrated in **Figure 1**. In **Figure 1A** the overall accuracy was plotted against the LC – both are derived from L and R (i.e., left and right ear accuracy). The regression (red line) was modeled with locally weighted scatterplot smoothing (LOESS), a nonlinear fitting procedure which ascribes a value “*y*” to a given value “*x*” on the basis of (weighted) local “*y*” values (Leask and Crow, 2006). Specifically, we used the Matlab (The MathWorks, Natick, MA, USA) function “rloess” (robust LOESS) with a span of 0.7 (cf. Hirnstein et al., 2010). In a second step (**Figure 1B**) the original data (red line) is plotted against reference models (blue lines) with near to zero correlations between L and R. The reference models were generated from the original data to ensure that the only difference between reference models and original data was the removed L–R-correlation: one side, say L, was displaced by one row such that L from participant 1 was matched with R from participant 2, and L from participant 2 with R from participants 3, etc., until finally L from participant 1839 was matched with R from participant 1. The mean and standard deviation of this displaced L is identical to the original L but the correlation between the displaced L and R is different to the correlation between the original L and R. The displacement was repeated 1838 times leading to 1838 different L–R pairs. As reference models, however, only those L–R pairs were chosen in which the correlation was *r* < 0.01 – thus effectively 0. The overall accuracy and the LC derived from these L–R pairs served as reference models. They were plotted alongside the original data and also modeled with LOESS (**Figure 1B**). To reveal the relationship between degree of lateralization and performance – controlled for interdependency between L and R – all reference models were subtracted from the original data (**Figure 1C**) and averaged to ease interpretation (**Figure 1D**): if the red mean subtraction line is above zero, performance is good – relative to a reference model in which interdependency has been removed. If the line is below zero, then performance is relatively poor and if the line is zero, then no meaningful interpretation of performance is possible. For further details we refer to Leask and Crow (1997, 2006).

## RESULTS

**FIGURE 2:**
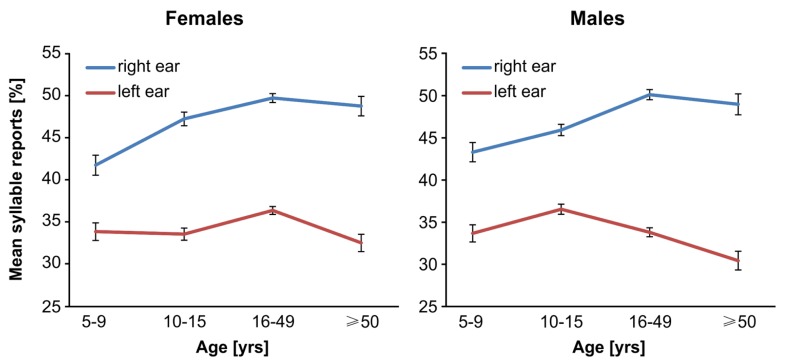
**Mean left and right ear reports (±**SEM) across sex and age. Both males and females in all age groups report more syllables from the right than the left ear. This right ear advantage is slightly stronger in males than females in all age groups except in early adolescents.

To demonstrate that the Bergen DL test shows the expected right ear advantage, left and right ear accuracy rates were subjected to a 2 × 2 × 4 mixed ANOVA with Ear (left, right) as within- and Sex, and Age (children, early adolescents, young adults, old adults) as between-participants factors. Participants reported more syllables from the right (47.0 ± 0.3%) than left ear (33.8 ± 0.3%) as indicated by a significant main effect Ear [*F*_(1,1831)_ = 547.99, *p* < 0.001, partial η^2^ = 0.23]. This right ear advantage became steadily larger with increasing age [interaction Ear by Age *F*_(3,1831)_ = 9.64, *p* < 0.001, partial η^2^ = 0.02], from childhood (right 42.5 ± 0.8%, left 33.7 ± 0.7%) via early adolescence (right 46.6 ± 0.5%, left 35.0 ± 0.5%) and younger adulthood (right 49.9 ± 0.4%, left 35.0 ± 0.4%) to older adulthood (right 48.9 ± 0.9%, left 31.5 ± 0.8%). Bonferroni adjusted *post hoc* tests revealed that compared to children early adolescents reported significantly more syllables from the right (*p* < 0.001) but not the left ear (*p* = 1). Younger adults had an even higher right ear accuracy than early adolescents (*p* < 0.001) but again left ear rates did not differ (*p* = 1). Older adults, however, had a lower left ear rate than younger adults (*p* < 0.001) but the right ear rates did not differ (*p* = 1). In all age groups, the right ear advantage was significant (all *p* < 0.001). The three-way interaction Ear by Sex by Age also became significant [*F*_(3,1831)_ = 3.86, *p* = 0.009, partial η^2^ = 0.01]. *Post hoc* tests revealed that female adolescents reported significantly more syllables from the right ear than female children (*p* < 0.01), while male children/early adolescents did not show such a rise (*p* = 1). The left ear reports did not change in both sexes (all *p* = 1). As a result female early adolescents showed a stronger right ear advantage than male early adolescents, whereas in all other groups males had a numerically stronger right ear advantage than females (see **Figure 2**). The right ear advantage was significant in both sexes in all age groups (all *p* < 0.01).

A main effect of Age [*F*_(3,1831)_ = 41.25, *p* < 0.001, partial η^2^ = 0.06] indicated that younger adults [*M* = 42.50% ± SEM** = 0.2] generally reported more syllables than older adults (40.2 ± 0.4%), early adolescents (40.8 ± 0.2%), and children (38.1 ± 0.4%). *Post hoc* tests were significant for all comparisons (all *p* ≤ 0.001) except for the difference between early adolescents and older adults (*p* = 0.993). The better overall accuracy in younger adults depended upon Sex [interaction Age by Sex with *F*_(3,1831)_ = 4.12, *p* = 0.006, partial η^2^ = 0.01]. While males obtained higher overall accuracy than females in childhood (males 38.5 ± 0.5%, females 37.8 ± 0.5%) and early adolescence (males 41.2 ± 0.3%, females 40.4 ± 0.4%), females reported more syllables correctly than males in younger (females 43.0 ± 0.02%, males 42.0 ± 0.3%) and older adults (females 40.6 ± 0.5%, males 39.7 ± 0.6%). However, none of these sex differences was significant after Bonferroni adjustment (all *p* ≥ 0.109).

Handedness was analyzed separately, since there were not sufficient non-right-handers (see **Table 1**) for including this variable in the ANOVA above. Non-right-handers were matched to right-handers on the basis of sex and age. A 2 × 2 ANOVA with Ear and Handedness as within- and between-participants factors, respectively, only revealed a significant main effect Ear [*F*_(1,324)_ = 78.56, *p* < 0.001, partial η^2^ = 0.20] with the expected right ear advantage (right 46.79 ± 0.7%, left 36.7 ± 0.6%). Neither the main effect Handedness nor the interaction Ear by Handedness reached significance (all *F* ≤ 1.10, *p* ≥ 0.295).

### THE RELATIONSHIP BETWEEN ASYMMETRY AND PERFORMANCE

#### Traditional approach

**FIGURE 3:**
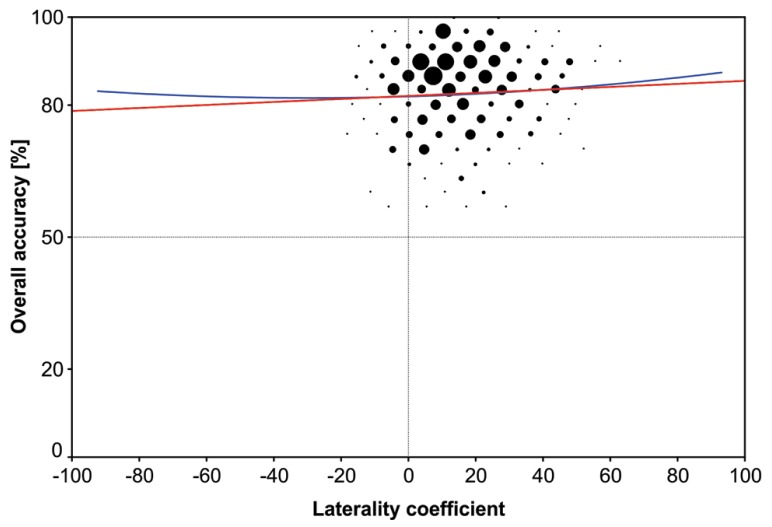
**Results of the traditional approach.** The bubble chart shows the linear (red line) and quadratic regression (blue line) between overall accuracy and degree of lateralization.

A statistically significant, positive correlation emerged between directional LC (preserving the direction of asymmetry) and overall accuracy [*F*_(1,1837)_ = 9.21, *p* = 0.002] showing that participants reported more correct syllables the more strongly their right ear advantage was (**Figure 3**). The correlation coefficient was rather small (*r* = 0.07) and accounted for 0.5% of the variance. The quadratic model also became significant [*F*_(2,1836)_ = 5.70, *p* = 0.003] suggesting that, in general, stronger ear advantage (regardless of its direction) was associated with higher performance. However, the explained variance was only marginally higher than in the linear model (*R*^2^ = 0.6%). The absolute LC and overall accuracy also showed a statistically significant but very small linear correlation (*r* = 0.06, *p* = 0.009) accounting for 0.4% variance. The same applies to the quadratic model [*F*_(2,1836)_ = 4.42, *p* = 0.012, *R*^2^ = 0.5%].

Finally, left and right ear accuracy rates were negatively correlated (*r* = -0.51, *n* = 1839, *p* < 0.0001). Thus higher right ear rates were associated with lower left ear rates.

#### LOESS approach

**FIGURE 4:**
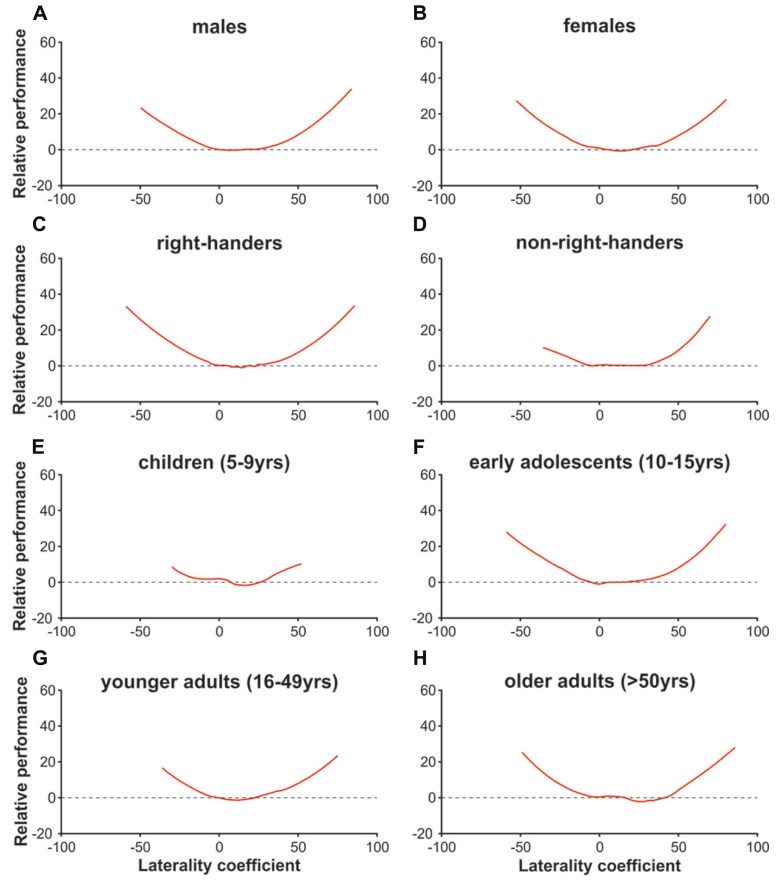
**The relationship between (relative) performance and degree of lateralization with the alternative approach in males (A)**, females **(B)**, right-handers **(C)**, non-right-handers **(D)**, children aged 5–9 **(E)**, early adolescents aged 10–15 **(F)**, younger adults aged 16–49 **(G)**, and older adults aged 50+ **(H)**.

**Figure 1D** shows a u-shaped relationship between asymmetry and performance across all participants. Similar to the traditional approach, the stronger the ear advantage (regardless of its direction) the more syllables were reported correctly. Relative performance declines as the ear advantage becomes smaller and is lowest at an LC of 11.52. **Figure 4** shows the asymmetry-performance relationship for females, males, right- and non-right-handers, children, early adolescents, younger adults, and older adults. The u-shaped curve was similar in all these groups: performance was lowest with a small right ear advantage (i.e., LC between 5 and 15) and steadily improved as the left or right ear advantage became stronger.

## DISCUSSION

The present study investigated how the degree of lateralization is related to overall accuracy in a verbal (consonant-vowel) DL task. Previous studies addressing the asymmetry-performance relationship were subject to interdependency issues of L and R scores. Moreover, the large sample size allowed exploring whether the asymmetry-performance relationship varies across sex, age, and handedness.

First of all, the results from the ANOVA confirmed the well-known right ear/left-hemispheric advantage for auditory speech processing (for review Bryden, 1988). This functional asymmetry was dependent upon age and sex, which is discussed in detail in Hirnstein et al. (2013). It should also be noted that the number of participants in the four age groups were different which means that the statistical power to detect effects is higher in early adolescents and younger adults group as compared to children and older adults. Nevertheless, the right ear/left-hemispheric advantage emerged, on average, across all participants and in all subgroups in accordance with the literature (Hugdahl, 2003). However, as can be seen in **Figure 3**, there was considerable interindividual variation with respect to whether a left or right ear advantage emerged and how strong this advantage was. The variability in the degree and direction of the ear advantage in our sample thus allowed us to examine whether DL performance depends on the strength and/or the direction of the ear advantage. The traditional approach of correlating the degree of lateralization with the overall accuracy revealed a significant quadratic model. That is, a u-shaped curve where individuals with stronger right and left ear advantages reported more syllables correctly. However, the explained variance of 0.6% was trivial. Since the bulk of participants had a right ear advantage, the correlation (linear model) also became significant. That is, overall accuracy increased as the right ear advantage increased, but again, the explained variance was low (0.5%) and the correlation coefficient of *r* = 0.06 was well below what is considered a small effect (*r* = 0.20; Cohen, 1988). The flat regression lines in **Figure 3** neatly illustrate how meager the asymmetry-performance relationship is, which merely reached significance because of the large sample size. The LOESS approach (Leask and Crow, 1997, 2006), however, revealed a marked u-shaped relationship across all participants (**Figure 1D**) confirming that stronger ear advantages result in better performance.

This u-shaped relationship was largely in alignment with previous investigations of the asymmetry-performance relationship in verbal DL tasks. Boles et al. (2008) used a consonant-vowel task similar to the Bergen DL task and found a positive correlation between *absolute* ear asymmetry and overall accuracy in right-handed adults. Thus, a stronger ear advantage was associated with higher accuracy, corresponding to the u-shaped curve observed in the present study. The follow-up study by Barth et al. (2012) found only trends for a positive correlation – presumably due to small sample size. For the same reason van Ettinger-Veenstra et al. (2010) might have failed with a sample size of *n* = 16 to find correlations between ear asymmetry and overall accuracy in the non-forced condition of the Bergen DL task.

Why was there such a considerable discrepancy between the traditional and the LOESS approach in the present study? Moreover, why did Boles et al. (2008) find a u-shaped asymmetry-performance relationship (similar to results of the LOESS approach reported here), although they used the traditional approach? The answer to these questions might lie in the response format of the Bergen DL task. As pointed out above, the task in the present study used a one-response paradigm. That is, participants reported *either* the left *or* the right ear stimulus. The advantage of such one-response paradigms is that it deals better with extremely high performances. For example, a participant with 100% overall accuracy could have either reported all stimuli from the left ear, all stimuli from the right ear, or 50% from each ear. Accordingly, the participant would be classified as strongly right-lateralized, left-lateralized or perfectly bilateral. In a two-response paradigm, however, participants with 100% accuracy in both the left and the right ear can only be classified as perfectly bilateral. Moreover, a one-response paradigm avoids confounding the reports by introducing a working memory component. If more than one answer is required, one syllable has to be kept active in the working memory buffer while the first syllable is reported. The disadvantage with one-response paradigms is that L and R scores are more likely to correlate negatively, increasing the problem of interdependency. In the present study, L and R scores were indeed negatively correlated (*r* = -0.51, *p* < 0.0001) and therefore the LOESS approach was crucial here. However, this does not mean that the LOESS approach should only be applied to one-response paradigms. It seems reasonable to assume that there are also high (positive) correlations between L and R in two-response paradigms, since participants with high accuracy in one ear/visual half-field typically also perform rather well on the contralateral side. For example, in our own word recognition and face discrimination task we found correlations between L and R scores of *r* = 0.60 (*n* = 229, *p* < 0.001) and *r* = 0.55 (*n* = 229, *p* < 0.001), respectively (Hirnstein et al., 2010). Interdependency issues are thus not limited to one-response paradigms and we therefore suggest employing the LOESS approach whenever substantial correlations between L and R scores arise.

The u-shaped pattern showing higher overall accuracy with increasing ear advantages can be seen – descriptively – in all sex, age, and handedness subgroups (**Figure 4**). Several studies investigated whether right-handers have higher cognitive abilities than, for instance, left-handers (Johnston et al., 2009; Nicholls et al., 2010; Mellet et al., 2013), but only few studies examined whether certain subgroups show a different *relationship* between lateralization and performance. Chiarello et al. (2009) found stronger correlations between verbal lateralization and reading performance in consistent as compared to inconsistent handers, but both groups showed positive correlations. Crow et al. (1998) reported that with increasing manual task asymmetry participants performed better in verbal tasks, but this (again) u-shaped relationship was similar in males and females. In accordance with these findings, the present study suggests that the u-shaped relationship between ear asymmetry and overall accuracy emerged in all subgroups. Although the findings of the present study are of descriptive nature, together with the previous findings it seems that, in general, the relationship between lateralization and performance shows little interindividual variation. Whether these findings can be generalized to other subgroups and non-verbal functions, however, needs to be clarified in future studies. We further hypothesized that groups with, on average, lower degrees of lateralization (females, non-right-handers, children) would, on average, obtain lower overall accuracy. This, however, was not necessarily the case. Indeed, right- and non-right-handers did not show any difference in the magnitude of the right ear advantage and also no difference in the number of reported syllables on average (missing main effect and missing interaction). Moreover, children showed the weakest right ear advantage and the lowest number of reported syllables on average. On the other hand, older adults showed a stronger right ear advantage than younger adults, but reported significantly fewer syllables in general. Likewise, female early adolescents were more strongly lateralized than male early adolescents but reported (non-significantly) fewer syllables in general (for more details Hirnstein et al., 2013).

Why is stronger ear asymmetry associated with higher accuracy? When two consonant-vowel stimuli are presented simultaneously, as in the present study, participants sometimes experience sound fusion, which makes it very difficult to correctly report stimuli. For instance, /ba/ and /ta/ are often merged into the sounds /pa/ or /da/ (Repp, 1977). In participants with a clear left or right ear preference, the signal strength for stimuli from the dominant ear seems to be consistently higher than for the non-dominant ear. As a result such fusions are less likely to occur and the error rate might be lower compared to participants without a clear ear preference where the signal from both ears is about equally strong (cf. Hirnstein, 2011). Although speculative at this stage, a reduced risk of such dichotic fusion errors in participants with a clear ear asymmetry might provide a reasonable explanation for the observed u-shaped curve. This also explains why asymmetry-performance relationships reported for verbal DL cannot be necessarily extrapolated to other tasks, processes, and sensory modalities, and thus might partly explain inconsistencies between studies, regardless whether the traditional or LOESS approach is used. For example, Hirnstein et al. (2010) found an *inverted* u-shaped relationship between degree of lateralization and accuracy in verbal and non-verbal visual half-field paradigms (i.e., word recognition and face discrimination). In this study, overall performance deteriorated as participants became more strongly lateralized. Thus, despite our expectation that asymmetry-performance relationship should not be different between sensory modalities, there may be different processes operated in visual as compared to auditory laterality tasks.

Several implications can be derived from previous studies together with the present findings. First, asymmetry-performance relationships are indeed task-dependent (Boles et al., 2008). As far as language is concerned, however, stronger lateralization seems to be associated with better performance in verbal abilities (Catani et al., 2007; Boles et al., 2008; Chiarello et al., 2009; Everts et al., 2009; van Ettinger-Veenstra et al., 2010; Barth et al., 2012). Second, the assumption that stronger brain asymmetry is generally beneficial, which has been reported especially in the animal literature (Güntürkün et al., 2000; Rogers et al., 2004), is not correct *per se*. As pointed out by Corballis (2005, 2006), both strong asymmetries as well as a more bilateral functional brain organization have advantages and disadvantages which need to be held in balance. Finally, the u-shaped (or inverted u-shaped) curves reported so far (Leask and Crow, 2006; Boles et al., 2008; Hirnstein et al., 2010) have their midpoints close to a lateralization degree of zero. Thus, participants with left- and right-hemispheric lateralization essentially show the same pattern: stronger asymmetry leads to better (or poorer) performance. This implies that *degree* of lateralization is far more important for performance than *direction* (i.e., whether a function is lateralized to the left or right hemisphere).

## CONCLUSION

Taken together, the findings of the present study showed that participants with stronger left or right ear advantage had higher overall accuracy in the verbal DL task. This u-shaped relationship between asymmetry and performance was similar across sex, age, and handedness and might result from fewer dichotic fusion errors in participants with clear ear asymmetries. In line with previous findings, the present study suggests that the degree of functional cerebral asymmetry is associated with the level of performance of a corresponding task. The hemisphere to which a function is lateralized, however, does not appear to be crucial. On the other hand, whether an asymmetric or symmetric brain organization is beneficial for performance depends on the particular task and the mental process(es) involved. Finally, the present study also emphasizes the importance of controlling for statistical interdependency between L and R scores when examining the asymmetry-performance relationship, particularly in one-response paradigms.

## AUTHOR CONTRIBUTIONS

Marco Hirnstein carried out the analyses. All authors contributed to the conception of the present study and participated in drafting the article.

## Conflict of Interest Statement

The authors declare that the research was conducted in the absence of any commercial or financial relationships that could be construed as a potential conflict of interest.
