# Erratum: How brain asymmetry relates to performance – a large-scale dichotic listening study

**DOI:** 10.3389/fpsyg.2014.00058

**Published:** 2014-01-31

**Authors:** Marco Hirnstein, Kenneth Hugdahl, Markus Hausmann

**Affiliations:** ^1^Department of Biological and Medical Psychology, University of BergenBergen, Norway; ^2^Department of Radiology, Haukeland University HospitalBergen, Norway; ^3^Division of Psychiatry, Haukeland University HospitalBergen, Norway; ^4^Department of Psychology, Durham UniversityDurham, UK

**Keywords:** hemispheric asymmetry, lateralization, dichotic listening, task-performance, sex, age, handedness, verbal abilities

Kenneth Hugdahl's second affiliation is Department of Radiology, Haukeland University Hospital, Bergen, Norway.

On page 1 the final sentence in the second column should read: “Moreover, individuals with *lower* degrees of language lateralization as determined with fMRI (van Ettinger-Veenstra et al., 2010) or magnetic resonance diffusion tensor imaging (Catani et al., 2007) performed better on tests assessing verbal abilities (van Ettinger-Veenstra et al., 2010) or verbal memory (Catani et al., 2007) than individuals with *higher* degrees of lateralization.”

On page 7 the final sentence of the first column should read: “For the same reason van Ettinger-Veenstra et al. (2010) might have failed with a sample size of *n* = 16 to find correlations between ear asymmetry and *behavioral language tests* in the non-forced condition of the Bergen DL task.”

On page 8 the final paragraph of the discussion should read: “As far as language is concerned, however, stronger lateralization seems to be associated with better performance in verbal abilities (Boles et al., 2008; Chiarello et al., 2009; Everts et al., 2009; Barth et al., 2012, *but see* Catani et al., 2007; van Ettinger-Veenstra et al., 2010).”
